# Sight impairment registration in Trinidad: trend in causes and population coverage in comparison to the National Eye Survey of Trinidad and Tobago

**DOI:** 10.1038/s41433-024-02943-3

**Published:** 2024-02-07

**Authors:** Shivaa Ramsewak, Frank Deomansingh, Blaine Winford, Debra Bartholomew, Vedatta Maharaj, Amandi Fraser, Deo Singh, Kenneth Suratt, Vrijesh Tripathi, Kevin McNally, Subash Sharma, Covadonga Bascaran, Samuel S. Ramsewak, Rupert R. A. Bourne, Tasanee Braithwaite

**Affiliations:** 1 The Medical Eye Unit, Ophthalmology Department, Guy’s and St Thomas’ NHS Foundation Trust, London, UK; 2 Today’s Optical, Trinidad, Trinidad and Tobago; 3 West Sussex General Practice rotation, London, UK; 4 Ophthalmology Department, Port of Spain General Hospital, Port-of-Spain, Trinidad and Tobago; 5 Richmond University Medical Center, Staten Island, NY USA; 6 Department of Optometry, The University of the West Indies, St Augustine Campus, St. Augustine, Trinidad and Tobago; 7 Caribbean Eye Institute, Valsayn, Trinidad, Trinidad and Tobago; 8 Trinidad and Tobago Blind Welfare Association, Port-of-Spain, Trinidad and Tobago; 9 Department of Mathematics and Statistics, The University of the West Indies, St Augustine Campus, St. Augustine, Trinidad and Tobago; 10 Low vision service, Ophthalmology, Kettering General NHS Foundation Trust, England, UK; 11 London School of Hygiene and Tropical Medicine, London, UK; 12 Faculty of Medical Science, The University of the West Indies, St. Augustine, Trinidad and Tobago; 13 Vision and Eye Research Institute, School of Medicine, Anglia Ruskin University, Cambridge, UK; 14 Department of Ophthalmology, Cambridge University Hospitals, Cambridge, UK; 15 School of Population and Life course Sciences, King’s College London, London, UK

**Keywords:** Vision disorders, Outcomes research, Epidemiology

## Abstract

**Background:**

Little was known about the population coverage and causes of sight impairment (SI) registration within the Caribbean, or the extent to which register studies offer insights into population eye health.

**Methods:**

We compared causes of SI registration in the Trinidad and Tobago Blind Welfare Association (TTBWA) register with findings from the 2014 National Eye Survey of Trinidad and Tobago (NESTT), and estimated registration coverage. Cross-sectional validation studies of registered clients included interviews, visual function and cause ascertainment in July 2013, and interviews and visual function in July 2016.

**Results:**

The TTBWA register included 863 people (all ages, 48.1%(*n* = 415) male) registered between 1951 and 2015. The NESTT identified 1.1%(75/7158) people aged ≥5years eligible for partial or severe SI registration, of whom 49.3%(*n* = 37) were male. Registration coverage was approximately 7% of the eligible population of Trinidad. Nevertheless, there was close agreement in the causes of SI comparing the register and population-representative survey. Glaucoma was the leading cause in both the register (26.1%,*n* = 225) and population-based survey (26.1%, 18/69 adults), followed by cataract and diabetic retinopathy. In the validation studies combined, 62.6%(93/151) clients had severe SI, 28.5%(43/151) had partial SI and 9.9%(15/151) did not meet SI eligibility criteria. SI was potentially avoidable in at least 58%(*n* = 36/62) adults and 50%(*n* = 7/14) children.

**Conclusion:**

We report very low register coverage of the SI population, but close agreement in causes of SI to a contemporaneous national population-based eye survey, half of which resulted from preventable or treatable eye disease.

## Introduction

Disease registers have widespread applications across medicine. The value of national sight impairment (SI) registers as a research tool has been recognised for over fifty years [1]. Register studies offer long-term epidemiological insights complementary to population-representative surveys, and can support public health planning and service quality improvement [2]. Register coverage of the SI population is challenging to estimate in the absence of population-based survey data, with few studies estimating coverage to be between 21% and 53% [3, 4]. We identified register studies from 21 predominantly high-income countries (Supplementary Table 1) [2–40]. These highlight differing epidemiological trends in incident registration and leading causes over time, by age group and by world region. For example, leading causes range from trachomatous complications in Oman in 2000 [3], and retinal genetic conditions relating to co-sanguinity in Kuwait [5, 6], to cortical, optic nerve, and congenital diseases including retinopathy of prematurity in childhood onset blindness [12, 16, 20, 24, 30, 40], to age-related but potentially preventable eye diseases [2, 7, 13], and to congenital/inherited eye diseases in more recent high-income country register studies [9, 22, 31, 39]. Studies on blindness registration in low-middle income countries are scarce, with only one prior study from Latin America and the Caribbean [2].

Trinidad and Tobago is a twin-island state in the West Indies with a population of 1.3 million, 95.5% of whom live in Trinidad. It is a high-income former British colony. The Ministry of Health oversee a public health system offering universal, free access to basic services including eye care, devolved to five regional health authorities [41]. The eye care system is pluralistic, with significant service contribution from the private sector [41]. The Institute for the Blind formed a register of blind individuals in Trinidad and Tobago in 1914, and continued to maintain this register after being renamed the Trinidad and Tobago Blind Welfare Association (TTBWA) in 1947. The TTBWA is a statutory non-profit organisation which functions under the aegis of the Ministry of Social Development and Family Services. Registration follows assessment by an ophthalmologist and facilitates application for a disability grant and social welfare, and access to a range of TTBWA support services (Supplementary Table 2).

This study had five aims: (1) To explore the trend in registration with the TTBWA; (2) to explore registered causes of SI; (3) to ascertain the proportion of patients on the register with partial and severe SI, and explore access to low vision services (validation study); (4) to compare causes in all ages, comparing the register, validation study, and contemporaneous population-based National Eye Survey of Trinidad and Tobago (NESTT, 2014) [42, 43]; and (5) to estimate registration coverage of the national population living with SI in 2016.

## Methods

### Part 1: the TTBWA register

We analysed a recently digitised copy of the TTBWA register in July 2013. Digitisation was noted to be incomplete for southern and eastern Trinidad and Tobago. In July 2016, we analysed the fully digitised Trinidad register, and excluded Tobago (paper register unavailable). We extracted data on region of residence, age at registration, sex, and cause of vision loss. The register did not include race/ethnicity, visual acuity (VA) or field, or category of vision loss.

### Part 2: cross-sectional validation study

In July-August 2013, we conducted a cross-sectional survey of all TTBWA clients on the digitised register residing in Northwest Trinidad, using a standardised recruitment approach (Supplementary Table 3). Clinical assessment by the NESTT survey team, followed the same methodology as the NESTT study, outlined in full previously [42]. In brief, we administered questionnaires including demographics, medical history, and low vision experience. We assessed visual function by assessing presenting and best-corrected uniocular distance VA, visual fields to confrontation, and with formal perimetry if VA was better than 6/24. The optometrist performed refraction and low vision assessment. The ophthalmologist examined the fundus using slit lamp biomicroscopy, following dilatation, to ascertain cause.

During the NESTT study (October 2013-November 2014), we informed all participants identified to have eligible SI how to contact the TTBWA, and outlined services available. In July-August 2016, we extended the cross-sectional survey of all TTBWA registered clients residing in Trinidad, including those newly registered since 2013, or who had not previously been contacted in 2013, using the same recruitment strategy (Supplementary Table 3), but offering a home-visit for assessment. The study doctor administered the same questionnaires and assessed presenting and pinhole uniocular distance VA, and visual fields to confrontation, to categorise SI, without detailed ocular examination for cause validation.

### Definition of partial and severe sight impairment (SI)

In the TTBWA validation study and the NESTT, we defined SI based on the category of vision impairment and field in the better-seeing eye. We defined severe SI as: a best-corrected visual acuity (BCVA) of LogMAR >1.30(Snellen 3/60); or BCVA 1.30 to 1.00(6/60) with a reduced field of vision; or a very severely reduced field [44]. We defined partial SI as: BCVA 1.30 to 1.00(6/60) with a full field of vision; BCVA 0.60(6/24) or worse, with moderate reduction of field of vision or with a central part of vision that is cloudy or blurry; or BCVA 0.48(6/18) or worse with a large part of field of vision missing, e.g. hemifield or substantial peripheral loss [44].

### Part 3: Comparison of causes of sight impairment (SI) to the 2014 National Eye Survey (NESTT) and estimated register coverage of the SI population

The sampling strategy for NESTT has been reported previously [42, 43]. In this study, we analysed data on the primary cause of best-corrected vision impairment <6/18 and visual field limitation in the better-seeing eye amongst participants aged 5 years and above. We assumed that NESTT participants were not eligible for registration if they had uncorrected refractive error, or cataract with BCVA in the better-seeing eye better than or equal to 6/60(LogMAR 1.00). If presenting VA < 6/18 and ≥6/60 and the person did not attend the regional clinic or have a domiciliary visit for best-corrected (or pinhole) acuity, fields and ascertainment of cause, we conservatively excluded the person from our definition of SI eligibility.

We used the mid-2016 population of Trinidad and Tobago aged 5 years and above (1,257,940), and the proportion of participants eligible for partial or severe SI registration in the NESTT sample aged 5 years and above, to estimate the number with SI in the national population. We reduced this number by 4.5% to estimate the number with SI in Trinidad alone (denominator). We estimated register coverage to be the percentage of clients on the TTBWA register in Trinidad in mid-2016 (numerator).

### Ethical and government approval

The study adhered to the tenets of the Declaration of Helsinki. Ethical approval was obtained from the Ethics Committees of the University of the West Indies (May 2012), the Ministry of Health of Trinidad and Tobago (May 2013) and Anglia Ruskin University (July 2013). Approval for the validation study extension to 2016 was obtained from the Ethics Committees of the University of the West Indies, Anglia Ruskin University, and from the Ethics Committee of The London School of Hygiene and Tropical Medicine (July 2016). We obtained written informed consent to participate, in the presence of a sighted family member, friend or independent advocate. We asked children aged 5-12 years and young people aged 13–17 years to sign separate assent forms, and consent was obtained from a legal guardian.

### Statistical methods

Statistical analyses were performed using standard statistical software (StataCorp.2023.Stata Statistical Software:Release 18.0.College Station,TX: StataCorp LP). We explored raw data on the register, and from the validation studies and NESTT sample, with simple descriptive statistics. The odds of responding to the validation study were explored with logistic regression (see Supplementary results). Single potential explanatory variables were considered one at a time. A multiple logistic regression model was estimated to control for the effects of potential explanatory and confounding variables on the odds of being a responder. For parameter estimation, global p-values were obtained using the likelihood ratio test (LRT). A p-value of 0.05 or lower was taken to be statistically significant.

## Results

### Part 1: analysis of the TTBWA Register in July 2013 and July 2016

**Fig. 1:**
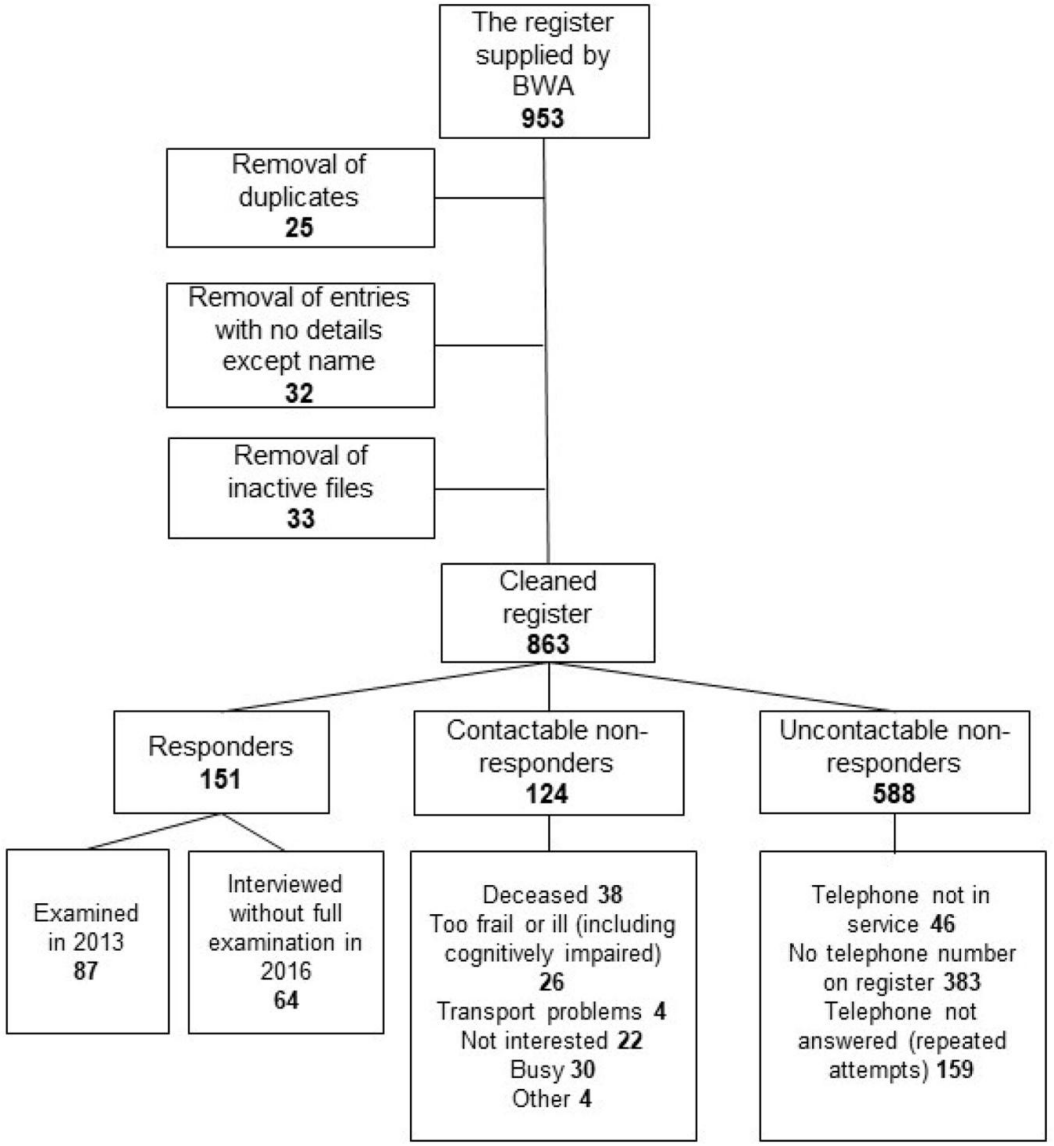
Flow diagram outlining Trinidad and Tobago Blind Welfare Association Register cleaning. Flow diagram outlining Trinidad and Tobago Blind Welfare Association Register cleaning undertaken in July 2013 and July 2016, and response rates to the validation study.

**Fig. 2:**
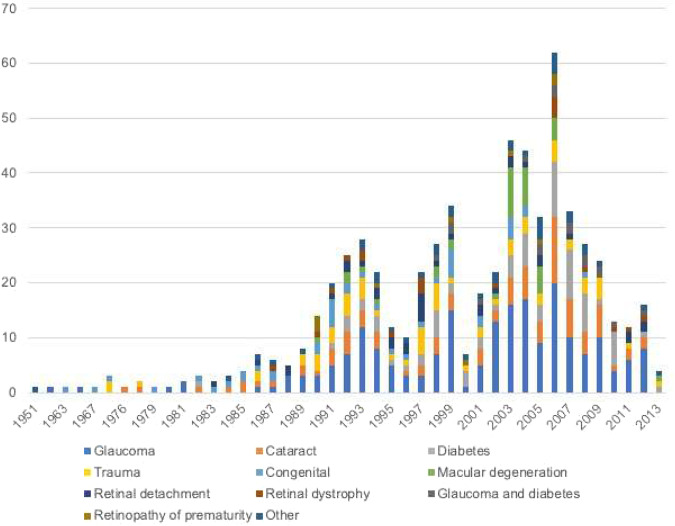
Bar chart illustrating new registrations with the Trinidad and Tobago Blind Welfare Association (TTBWA) per year, from 1951 to 2013. **Br**eakdown of new registrations by cause was not yet available for 2014 and 2015 at the time of data extraction, and these years are not shown.

After cleaning and de-duplication, there were 726 clients on the electronic register in July 2013, and 863 in July 2016 (See Fig. 1). Figure 2 illustrates new registrations by year and by cause between 1951 and 2013. Cause breakdown for new registrations in 2014 and 2015 was not available at the time of data extraction. Table 1 illustrates the 3-year growth rate in registrations from 2013 to 2016, broken down by region within Trinidad, and by new registrations (16.8%, *n* = 23) versus pre-existing paper registrations being digitised (83.2%, *n* = 114). This revealed an overall growth rate over the interval of 15.7%, and 3.2% growth in new registrations, with six-fold regional variation in the latter, ranging from 0.5% in the Northwest to 6.3% in the Central region. This may reflect historically higher registrations in the Northwest (near TTBWA headquarters in the capital city, Port-of-Spain) or via the regional office in the Southwest, and improved awareness of the TTBWA in the North Central and Eastern regions following NESTT.

**Table 1:** Comparison of TTBWA register in July 2013 and July 2016, revealing growth in registrations, driven by digitisation of the paper record and by new incident registrations.

Region	Total n	New registrations due to location of further paper-based records (with 3-year growth rate), n(%)	New registrations (with 3-year growth rate), n(%)	Percentage registered sight impaired compared to mid-year regional population size (all ages) n/N(%)
Northwest				
2013 Register	206			206/297,604 (0.07)
2016 Register	281	68 (33.0)	7 (0.5)	281/300,565 (0.09)
Change n(%)	75 (36.4)			
Central				
2013 Register	175			175/246,662 (0.07)
2016 Register	222	36 (20.6)	11 (6.3)	222/249,116 (0.09)
Change n(%)	47 (26.9)			
Eastern				
2013 Register	50			50/111,266 (0.04)
2016 Register	61	8 (16.0)	3 (6.0)	61/112,373 (0.05)
Change n(%)	11 (22.0)			
Southwest				
2013 Register	285			285/624,700 (0.05)
2016 Register	289	2(0.7)	2 (0.7)	289/630,915 (0.05)
Change n(%)	4 (1.4)			
Address not listed				
2013 Register	10			
2016 Register	10	0	0	
Change n(%)	0			
TOTAL				
2013 Register	726			726/1,340,557 (0.05)
2016 Register	863	114 (15.7)	23 (3.2)	863/1,353,895 (0.06)
Change n(%)	137 (18.9)			

^*^Regional population proportions from latest (2011) Census: 22.2% Northwest, 18.4% North Central, 8.3% Eastern, 46.6% South West and 4.5% Tobago, applied to 2013 mid-year population (1,340,557) and 2016 mid-year population (1,353,895). Source: https://cso.gov.tt/subjects/population-and-vital-statistics/population/

### Characteristics of clients on the TTBWA register

The mean age of 863 clients on the 2016 TTBWA Register was 62.1 years (standard deviation, sd 20.7), and ranged from 2 to 107 years, indicating that some uncontactable people were deceased. 48.1% (*n* = 415) clients were male. An address was available for 98.8% (*n* = 853) and one or more telephone numbers were available for 55.4% (*n* = 478). The mean age at registration was 47.6 (sd 22.2) years. The regional distribution of clients closely resembled the distribution of the general population in the 2011 Census (see Table 1).

### Causes of blind registration

Cause of SI was documented for 81.8% (706/863), and based on provision of a letter from an Ophthalmologist to the TTBWA for each registered client. Leading causes included glaucoma (*n* = 225, 26.1%), cataract (*n* = 99, 11.5%), diabetes (*n* = 82, 9.5%), and trauma (*n* = 67, 7.8%), followed by congenital causes (*n* = 48, 5.6%), macular degeneration (*n* = 46, 5.3%), retinal detachment (*n* = 36, 4.2%), retinal dystrophy including retinitis pigmentosa (*n* = 23, 2.7%), diabetes and glaucoma combined (*n* = 18, 2.1%) and retinopathy of prematurity (*n* = 11,1.3%) (Table 2). Other causes (23.9%), each accounted for ≤1%, and included intracranial pathology, optic nerve pathology, corneal pathology, infectious eye disease, inflammatory eye disease, ocular cancer, drug toxicity, vitamin A deficiency, amblyopia, and other rare causes.

**Table 2:** The primary cause of best-corrected partial and severe sight impairment, comparing the 2016 TTBWA register, TTBWA validation study in 2013 (in adults and children), and in 2016, and the 2014 National Eye Survey of Trinidad and Tobago sample.

Vision category and primary cause	TTBWA (all ages)	TTBWA validation study 2013 Adults	TTBWA validation study 2013 Children 5-17 y	TTBWA study 2016 Adults	NESTT 2014 Adults – All SI ≥ 18 y	NESTT 2014 Children – All SI 5-17 y
N (%)	863	71 (100)	16 (100)	64 (100)	69/5718 (0.75%)	6/1440 (0.42%)
SSI	NR	43 (60.6)	10 (62.5)	40 (62.5)	43 (62.3)	3 (50)
SI	NR	19 (26.8)	4 (25.1)	20 (31.3)	26 (37.7)	3 (50)
Not eligible	NR	9 (12.7)	2 (12.5)	4 (6.3)	NA	NA
N verified with SI	NR	62 (87.3%)	14 (87.5%)	60 (93.8%)	69 (100)	6 (100)
Glaucoma	225 (26.1)	14(22.6)	1 (7.1)	15 (25.1)	18 (26.1)	0
Cataract	99 (11.5)	1 (1.6)	0	3 (5.0)	12 (17.4)	0
Diabetes-related	82 (9.5)	5 (8.1)	0	8 (13.3)	14 (20.3)	0
Trauma	67 (7.8)	4 (6.5)	0	7 (11.7)	1 (1.5)	0
AMD	46 (5.3)	1 (1.6)	0	1 (1.7)	5 (7.3)	0
Congenital	45 (5.2)	9 (14.4)	4 (28.5)	1 (1.7)	4 (5.8)	1 (16.7)
Congenital cataract	3 (0.4)	2 (3.2)	2 (14.3)	0	0	0
Retinal detachment	36 (4.2)	1 (1.6)	2 (14.2)	3 (5.0)	0	0
Retinal dystrophy	23 (2.7)	5 (8.1)	0	6 (10.0)	2 (2.9)	0
Diabetes + Glaucoma	18 (2.1)	0	0	0	0	0
ROP	11 (1.3)	3 (4.8)	1 (7.1)	5 (8.3)	0	0
Optic nerve-related	11 (1.2)	3 (4.8)	2 (14.3)	0	2 (2.9)	1 (16.7)
Intracranial/ cancer	9 (1.0)	3 (4.8)	1 (7.1)	3 (5.0)	0	0
Ocular cancer	8 (0.9)	0	0	0	0	0
Other or multiple	8 (0.9)	5 (8.1)	0	0	1 (1.5)	2 (33.3)
Uveitis	7 (0.9)	0	1 (7.1)	2 (3.4)	1 (1.5)	0
Corneal pathology	5 (0.6)	2 (3.2)	0	1 (1.7)	1 (1.5)	0
Refractive/ amblyopia	3 (0.4)	1 (1.6)	0	2 (3.4)	2 (2.9)	0
Surgical complications	NR	3 (7.1)	3 (4.8)	0	1 (1.5)	0
Unknown	157 (18.2)	0	0	3 (5.0)	3 (4.4)	2 (33.3)

SSI Severe Sight Impairment = Best-corrected visual acuity(BCVA) of LogMAR >1.32, or >1.00(6/60) and ≤1.32 with reduced field of vision, or very severe reduction of visual field; SI Sight Impairment = >1.00 (6/60) and ≤ 1.30(3/60), or BCVA of LogMAR 0.60(6/24) or worse, with a moderate reduction of field of vision or with a central part of vision that is cloudy or blurry, or BCVA of LogMAR 0.48 or worse (up to 6/18), with a large part of field of vision missing, e.g. hemifield or substantial peripheral loss.

*KEY* AMD age-related macular degeneration, *NA* not applicable, *NR* not recorded, *ROP* retinopathy of prematurity.

### Part 2: response rate to TTBWA validation survey

In July 2013, we endeavoured to contact 206 TTBWA clients residing in the Northwest of Trinidad. We identified that 14 clients were deceased and 46 had incorrect or missing contact details. Of the remaining 146, we contacted 117(80.1%), of whom 74 (50.7% response rate) attended the research clinic for assessment, including three people aged under 18 years. In addition, of the 17 students enroled at the School for the Blind in July 2013, 13 (76.5%) attended.

In 2016, we endeavoured to contact all new (*n* = 22) and previously uncontacted clients by telephone. 588 clients were not contactable. Of the 188 clients with contactable telephone numbers, 38 were deceased. Of the 150 contactable clients, 64 (42.7%) consented to participate in interviews and assessment of VA and confrontation field.

In total, the contact rate across both validation studies was 31.9% (275/863). Excluding 4.4% (*n* = 38) confirmed deceased, 63.7% (151/237) contactable clients agreed to participate. Reasons offered for non-response are detailed in Fig. 1. To explore client characteristics, we combined data from the 2013 and 2016 validation studies in the remainder of this analysis.

Responders (*n* = 151) and non-responders (*n* = 712) differed significantly in having younger age, fewer years since registration, and region of residence, but not in sex, marital status or cause of blindness (Supplementary Table 4). Demographics of TTBWA clients were similar to the 2011 Population Census data in terms of gender and age group distribution, but differed in ethnicity, religion and marital status.

### Characteristics of TTBWA clients participating in validation study

In the 2013 validation study of adults, 60.1% (43/71) had severe SI, 26.7% (19/71) had partial SI and 12.7% (9/71) were not eligible. In the 2016 validation study of adults, 62.5% (40/64) had severe SI, 31.3% (20/64) had partial SI and 6.3% (4/64) were not eligible. Amongst those ineligible for SI registration, 5 had monocular blindness, and the remaining 8 had VA better than 6/60 without field loss. These people were removed from further analysis. The most frequent causes of blindness in adults included glaucoma, diabetic retinopathy, trauma, retinal dystrophy, and congenital causes (Table 2). Examples of potentially avoidable causes of SI are detailed in Supplementary table 5. Clients were asked, “*is there anything you would like to change about the current eye care system in Trinidad*” and a narrative summary of answers is provided in Supplementary Table 6.

The median age of adults with SI was 54 years (IQR 42–66, range 18–97) and 43.8% (*n* = 53/121) were male. 61.2% (74/121) reported African ancestry and 23.1% (28/121) reported East Indian ethnicity, with 15.7% (19/121) reporting ‘other’. Literacy was reported by 81.8% (88/121). Employment status was reported to be: employed, 28.9% (35/121); retired, 24.0% (29/121); disabled, 22.3% (27/121); student, 7.4% (9/121); home duties 5.0% (6/121); unemployed 5.8% (7/121); and not stated/other 6.6% (8/121). A prior low vision assessment was reported by 25.6% (*n* = 31/121). Ability to read Braille was reported by 35.5% (43/121) and ability to read large print was reported by 46.3% (56/121). 5.8% (7/121) reported use of a handheld magnifier, 1.7% (2/121) used a stand magnifier, 16.5% (20/121) used a spectacle magnifier, 5.0% (6/121) used magnification software, 63.6% (77/121) used a white cane and 1.7% (2/121) had a guide dog.

Amongst children <18 years, 62.5% (10/16) had severe SI, 25.1% (4) had partial SI and 12.5% (2) were not eligible. The reasons for non-eligibility included one child who had never owned spectacles and had presenting VA 6/60, which corrected to 6/6 following refraction; and one child who had recently had surgery and secondary lens implantation for congenital cataract, with BCVA 6/18 and no field loss. Median age was 13.5 years (IQR10.5-15, range 6–17), and 62.5% (10/16) were male. Leading causes of SI included causes congenital (28.5%, 4/14), congenital cataract (14.3%, 2/14), optic atrophy (14.3%, 2/14) and retinal detachment (14.2%, 2/14, secondary to neuroblastoma, and to blunt trauma), with one case each (7.1%) of glaucoma, intracranial pathology, retinopathy of prematurity, and uveitis. 81.3% (13/16) attended the residential School for the Blind, 2 (12.5%) attended primary and secondary school, and 1 (6.3%) did not attend school on account of neurological comorbidity. Ability to read Braille was reported by 56.3% (9/16), and ability to read large print was reported by 43.7% (7/16). 25.0% (4/16) reported use of a handheld magnifier, one child (6.3%) used a stand magnifier, and 31.3% (5) used a white cane.

We considered vision loss to have been potentially avoidable in at least 58% (*n* = 36/62) adults and 50% (*n* = 7/14) children.

### Part 3: Comparison to the National Eye Survey of Trinidad and Tobago (NESTT) and estimate of register coverage

In NESTT, the crude prevalence of SI was 69/5718 (0.75%) amongst adults ≥18 years, and 6/1440 (0.42%) amongst 5 to 17-year-olds. Causes are presented in Table 2. Similar to the TTBWA study, NESTT identified leading causes of SI in adults to be glaucoma (26.1%, *n* = 18), diabetic eye disease and associated complications (20.3%, *n* = 14), cataract causing BCVA < 6/60 (17.4%, *n* = 12), AMD (7.3%, *n* = 5) and congenital abnormality (5.8%, *n* = 4). Less common causes (each 2.9%, *n* = 2) included optic nerve pathology, retinal dystrophy and amblyopia, and 1 participant (1.5%) had each of: the chronic sequelae of uveitis, corneal pathology, trauma, and surgical complications. Seven people with presenting VA < 6/18 did not attend for detailed examination and were excluded.

The NESTT conservatively identified 1.05% (75/7158) participants aged 5 years and above with SI eligible for registration. Extrapolating to the 2016 mid-year national population aged 5 years and above (*n* = 1,257,940) we estimate that there were 13,180 people with eligible SI. Of these, we estimate 12,587 lived in Trinidad, yielding a TTBWA register coverage in 2016 of 6.9% (863/12,587). Furthermore, in 2016 we found that only 22 new cases had been added to the register in 3 years, and following the NESTT, indicating ongoing low registration uptake, in spite of the population sensitisation relating to the eye survey undertaken in the national media, and NESTT identification of unregistered but eligible people with SI.

## Discussion

Glaucoma was the leading cause of registration in Trinidad, with remarkably close agreement across the TTBWA register data (26.1%), TTBWA 2013 validation study in adults (22.6%) and NESTT survey in adults (26.1%). There was close agreement in other causes of SI, over fifty percent resulting from potentially avoidable or treatable eye diseases, including diabetic retinopathy and cataract. Whilst this aligns with another study from Belize [2], it highlights critical need for eye care system strengthening.

Whilst our study provided reassuring validation for blind register studies as a proxy for research into the causes of SI, the low estimated population coverage of 7% highlights substantial risk of bias in using register studies to make epidemiological inferences. Whilst registration coverage is believed to be fair in other high-income countries like England (48%) [45], there is no direct evidence for this on account of the lack of nationally-representative population-based survey data, and registration being a voluntary opt-in process for affected individuals. A study in the Republic of Ireland found that only 43% of eligible people were registered [22]. Strategies to improve registration coverage include providing comprehensive information on the process and benefits of SI registration to eye care providers and patients, reminders from professional bodies, and public awareness campaigns [46]. Differences in register coverage may also relate to differing financial concessions offered between countries, including access to free sight tests, help with healthcare costs, discounted or free travel, tax allowances and support for claims for welfare benefits, many of which were not available in Trinidad and Tobago in 2014 (See Supplementary Table 7 for comparison of benefits in Trinidad and Tobago to UK [44], Massachusetts USA [47], and Israel [48]).

Our narrative literature review (Supplementary Table 1) identified substantial variation in SI registration eligibility criteria internationally, making direct comparison of the epidemiology and causes of SI challenging. We identified that the most frequently studied registers are those of the UK [4, 23–33], and Israel [34–40]. In the UK, registration is voluntary and eligibility criteria, though enshrined in law, were historically vague [23]. Unsurprisingly, a study in Denmark highlighted that statistics on blindness were sensitive to the eligibility criteria used [17].

Our study highlights unmet need in Trinidad and Tobago for: definition of SI registration eligibility and the legal basis for registration; a more robust registration process; and for future capture of both VA and visual field in each eye to specify category of SI, in line with the majority of other registers internationally (Supplementary Table 1). Our validation study (combining data from 2013 and 2016) found that 9.9% of TTBWA clients would not meet the legal criteria for SI registration in the UK. In many countries, registers serve a legal function, qualifying individuals to receive social and financial support, or directing them to education and rehabilitation services. Barriers to registration may include lack of understanding about the process amongst eye care providers, with uncertainty around when best to certify patients (in the context of ongoing clinical management and uncertain visual prognosis), and eye care providers viewing certification as the ‘final stage’ in treatment [49].

This study had multiple limitations, including some inherent to the TTBWA register. The register had not been actively maintained in terms of contact details, it had not been cleaned to remove deceased persons and duplicates, and there was missing data. We were only able to include the Trinidad register in this study, because the paper-based Tobago register, covering 4.5% of the total population, had not been digitised in July 2016. Furthermore, this study ascertained that there was no standardised form to support the registration process and acquisition of high-quality data, nor documentation of monocular or binocular vision level. We used a telephone contact approach, but only 56% of the database had telephone numbers, and resources did not permit attempted postal contact. The relatively low contact rate of 31.9% in this study, and some significant differences between responders and non-responders, indicated that a significant proportion of ‘no contacts’ may have emigrated or died.

## Recommendations

Practical guidance for establishing and maintaining a patient register has been outlined previously [50]. Recommendations include identifying and engaging with relevant stakeholders, including clinicians, patients and industry. Identifying goals for future data analysis and research can be useful in prioritising essential data fields to capture. We recommend introduction of a standardised SI certification form in Trinidad and Tobago, including local clarification on category of SI and legal standards for registration, and standardised terminology and codes (e.g. International Classification of Disease version 11) for cause documentation. We also recommend use of software to support secure electronic data storage and to facilitate automated annual summary reports. We recommend promoting benefits of registration through a public awareness campaign to enhance TTBWA registration uptake, with focus on reducing stigma around SI. Stakeholder engagement and further research would be valuable to understand current barriers to registration, with focus on currently under-represented groups.

## Conclusions

This study illustrates the value of surveying a national blind register in parallel to a national population-based eye survey, and illustrates the limited extent to which the former serves as useful proxy for the epidemiology of SI. In Trinidad, we found low population coverage of the register, but close agreement in leading causes of SI to the contemporaneous population-based survey in 2014. Further research is needed internationally to understand barriers to SI registration, and perceived and actual benefits of registration.

## Summary

### What was known before

- Registry studies on the sight impaired have been reported by at least 21 countries in the last 50 years, predominantly in high-income regions, with no previous studies in the Caribbean.
- We identified no registry studies conducted in parallel with a national population-based eye survey to explore coverage of eligible population by registration and degree of agreement on cause.

### What this study adds

- Across the 35-year period of registry data (1980 to 2015), leading causes of sight impairment (all ages) in Trinidad included glaucoma(*n* = 225, 26.1%), cataract(*n* = 99, 11.5%), diabetic retinopathy(*n* = 82, 9.5%), and trauma(*n* = 67, 7.8%), followed by congenital causes(*n* = 48, 5.6%), macular degeneration(*n* = 46, 5.3%), retinal detachment(*n* = 36, 4.2%), retinal dystrophy including retinitis pigmentosa(*n* = 23, 2.7%), diabetes and glaucoma combined(*n* = 18, 2.1%) and retinopathy of prematurity (*n* = 11,1.3%). These causes were similar to findings from the contemporaneous national population-based eye survey.
- An estimated 7% of the population with sight impairment were known to the register in 2016, highlighting that registry studies may be at considerable risk of bias.
- Potentially preventable, treatable and curable eye diseases remained leading causes of sight impairment in 2016, highlighting unmet need for eye care system strengthening in Trinidad and Tobago.

### Supplementary information

Supplementary Table 1

Supplementary Tables 2–7

## Data Availability

Further analyses on the unpublished data set are in progress with manuscripts in preparation. Data access is governed by the NESTT Steering Committee, chaired by a representative of the Faculty of Medical Science at the University of the West Indies (St Augustine, Trinidad) and academic partners in the Vision and Eye Research Unit at Anglia Ruskin University (Cambridge, UK), and The School of Life Course and Population Sciences, King’s College London.
