# Supplementary material for: Sight impairment registration in Trinidad: trend in causes and population coverage in comparison to the National Eye Survey of Trinidad and Tobago
Source: Eye (Lond). 2024 Feb 7;38(11):2134–42. doi: 10.1038/s41433-024-02943-3 (PMC11269719; doi:10.1038/s41433-024-02943-3)
Supplement: Supplementary file 1 — Supplementary Table 1 [file 41433_2024_2943_MOESM1_ESM.docx]

**Supplement**

**Table 1: Summary of Blind Registry Studies, by Global Burden of Disease (GBD) Super region**

| **Country and citation** | **Citation** | **Study Year** | **Epidemiologicestimate of blindness +/-registry coverage** | **N (age group)** | **Data variables included in register** | **Criteria for registration** | **Leading causes of blindness** |
| --- | --- | --- | --- | --- | --- | --- | --- |
| Belize | Baxter SLW, R.P.; Musa, J.M.; Patel, D. Blindness Registers as Epidemiological Tools for Public Health Planning: A Case Study in Belize. *Epidemiology Research International.* 2014:1-8 | 2009 | Not stated | 1194 (all ages) | Sex, age, district of residence, visual acuity, level of visual impairment, extent of rehabilitation needed, primary cause of registration | Best-corrected visual acuity (BCVA) of 20/200 or less in both eyes and determination by the referring ophthalmologist of no benefit from further medical treatment or surgery | Cataract (39.6%), glaucoma (20.8%), diabetic retinopathy (10.2%), and childhood blindness (9.4%) |
| **GBD Region: North Africa and Middle East** | | |  |  |  |  |  |
| Kuwait | Al-Merjan JI, Pandova MG, Al-Ghanim M, Al-Wayel A, Al-Mutairi S. Registered blindness and low vision in Kuwait. *Ophthalmic epidemiology.* 2005;12(4):251-257. | 2004 | Incidence 10.0 per 100,000 person-years | 412 between 2000-2004 (all ages) | Demographics, date of registration, cause of blindness, visual acuity, visual field | Visual acuity 6/60 or less in the better eye with best possible correction or a visual field less than 20 degrees around the central fixation point. | Retinitis pigmentosa, congenital anomalies, optic atrophy |
| Kuwait | Pandova MG, Al-Merjan JI, Sadeq NA. Registered blindness in Kuwait - 15 years of dynamic changes. *Ophthalmic epidemiology.* 2019;26(2):75-83. | 2014 | Incidence 17.59 per 100,000 (males) and 14.57 per 100,000 (females) | 421 males, 279 females (all ages) | Demographics, cause, visual acuity, visual field, family history | Best-corrected visual acuity 6/60 (0.1) or less or visual field constriction to 20 degrees or less in their better eye | Retinitis pigmentosa, glaucoma, diabetic complications |
| Oman | Khandekar R, Al Harby S. National Register for the Blind: a tool for health programme management. *Eastern Mediterranean health journal = La revue de sante de la Mediterranee orientale = al-Majallah al-sihhiyah li-sharq al-mutawassit.* 2006;12(1-2):170-177. | 2000 | 17,000 persons in Oman estimated to be blind yielding 20.7% coverage | 3525 (all ages) | Demographics, visual acuity, visual field, type of visual disability, cause of blindness | Visual acuity was < 3/60 in the better eye or the person had a correspondingly compromised field of vision | Trachomatous corneal opacity and phthisis bulbi, glaucoma, aphakia |
| **GBD Region: High Income** | | |  |  |  |  |  |
| Canada  Canada | Macdonald AE. Causes of Blindness in Canada: An Analysis of 24,605 Cases Registered with the Canadian National Institute for the Blind. *Canadian Medical Association journal.* 1965;92:264-279 | 1963 | Prevalence 131 per 100,000 | 24,605 (all ages) | Demographics, site and type of blindness, cause of blindness, age at onset, age at registration | VA in both eyes with proper refractive lenses 6/60 or less with Snellen chart or equivalent, or if the greatest diameter of the visual field in both eyes is less than 20 degrees. | Cataract (>15%), glaucoma (10%), malignant myopia (9%), pthisis bulbi (8%), corneal lesions (6%). In this study, 32% of the blindness was due to prenatal causes. |
|  | Pearce WG. Causes of blindness in children. 1046 cases registered with the Canadian National Institute for the Blind 1970-1973. *Canadian journal of ophthalmology Journal canadien d'ophtalmologie.* 1975;10(4):469-472 | 1973 | Not stated | 1046 children newly registered 1970-1973 (<20 years) | Demographics, diagnosis | VA in both eyes with proper refractive lenses is 6/60 or less with Snellen chart or equivalent, or if the greatest diameter of the visual field in both eyes is less than 20 degrees. | Cataract (13%), optic atrophy (12%), nystagmus (10%); retrolental Fibroplasia (6%) |
| Japan | Morizane Y, Morimoto N, Fujiwara A, et al. Incidence and causes of visual impairment in Japan: the first nation-wide complete enumeration survey of newly certified visually impaired individuals. *Japanese journal of ophthalmology.* 2019;63(1):26-33 | 2016 | Not stated | 12,505 adults years newly certified in 2015 (> 18 years) | Demographics, cause of blindness, visual impairment grade | Criteria as specified in the Act on Welfare of Physically Disabled Persons | Glaucoma (28.6%), retinitis pigmentosa (14.0%), diabetic retinopathy (12.8%), macular degeneration (8.0%) |
| Singapore | Lim KH. Registration of new blindness in Singapore for 1985-1995. *Singapore medical journal.* 1998;39(3):104-106. | 1995 | Not stated | 1248 (all ages) | Demographics, cause of blindness | Not reported | Retinal diseases (57.6%), congenital and developmental disorders (14.7%), optic atrophy (9.3%), glaucoma (8.6%), minor causes (1%). |
| Taiwan | Tsai IL, Woung LC, Tsai CY, et al. Trends in blind and low vision registrations in Taipei City. *European journal of ophthalmology.* 2008;18(1):118-124. | 1995 to 2004 | Blind registrations 2004 (per 100,000): 6.69 males, 6.01 females,  Low vision registrations 2004 (per 100,000): 12.90 males, 11.10 females | 2912 of which 44.2% blind (all ages) | Demographics, cause of blindness | Low vision: visual acuity worse than 6/18 (20/60) to a lower limit of 3/60 (20/400). Blindness: visual acuity worse than 3/60 (20/400) in the better eye with best possible correction. | Glaucoma, optic neuropathy, diabetic retinopathy, retinitis pigmentosa, age-related macular degeneration, myopic macular degeneration |
| New Zealand | Chong C, McGhee CNJ, Dai SH. Causes of childhood low vision and blindness in New Zealand. *Clin Exp Ophthalmol.* 2019;47(2):165-170 | 2018 | Prevalence 1.1 per 1,000. prevalence of childhood blindness and low vision was 0.05% and 0.06% | 1000 children (<23 years) | Demographics, cause of blindness, comorbidities, family history, visual acuity, visual field, examination findings | Best-corrected visual acuity </=6/18, or binocular visual field <10 degrees | Cortical visual impairment (31.5%), optic nerve atrophy (16.5%), optic nerve hypoplasia (9.0%). Main preventable causes of blindness were neonatal trauma/asphyxia (31.5%), retinopathy of prematurity (18.2%), non-accidental injury (10.3%). |
| Australia | Cooper RL. Blind registrations in Western Australia: a five-year study. *Australian and New Zealand journal of ophthalmology.* 1990;18(4):421-426 | 1984-1988 | 94 to 103 per 100,000 per year, across the 5 years | 7,256 (all ages) | ICD code (1979), Braille certificate number, demographics | Visual acuity less than 6/60 in both eyes or less than 10 degrees of functional visual field if central vision better than 6/60 in the better eye | Age-related maculopathy 51.9%, primary open-angle glaucoma 12.4%, optic atrophy 4.2%, macular scars, holes and atrophy 3.1%, diabetic retinopathy 3.1%, retinal detachment 3.0%, retinal vein occlusions 2.5% followed by cataract and pigmentary retinopathy at 2.0% each |
| Australia | Yong VK, Morgan WH, Cooper RL, et al. Trends in registered blindness and its causes over 19 years in Western Australia. *Ophthalmic epidemiology.* 2006;13(1):35-42 | 1984-2002 | Mean incidence of blindness over the 19 years:12.7/100,000/year | 3,852 (all ages) | ICD code (1979), Braille certificate number, demographics | Visual acuity less than 6/60 in both eyes or less than 10 degrees of functional visual field if central vision is better than 6/60 in the better eye | Age-related macular degeneration 53.7%, glaucoma 10.7%, optic neuropathy 4.8%. |
| Denmark | Rosenberg T. Visual impairment in Danish children 1985. *Acta Ophthalmol (Copenh).* 1987;65(1):110-117 | 1985 | Incidence: 14 per 100,000 children per year (<18 years) | 150 | Demographics, ophthalmological diagnosis, year of birth, aetiology, visual acuity, birth weight, any additional handicap | Visual acuity 0.3 or lower | Optic nerve atrophy, amblyopia, tapetoretinal degeneration, congenital nystagmus |
| Denmark | Riise R. Nordic registers of visually impaired children. *Scand J Soc Med.* 1993;21(2):66-68 | 1992 | Prevalence: 41 per 100,000 children <17 years | 2527 children in Denmark, Norway, Iceland, FInland | Sex, year of birth, year of registration, classification of visual impairment, ocular diagnosis, systemic diagnosis, aetiology and evt. additional impairments | Best corrected visual acuity in the best eye less than 6/18 or visual field less than 10 degrees around fixation | Optic nerve atrophy, cerebral amblyopia, retinopathy of prematurity |
| Denmark | Rosenberg T, Klie F. Current trends in newly registered blindness in Denmark. *Acta ophthalmologica Scandinavica.* 1996;74(4):395-398. | 1993 | Not stated | 1585 newly registeredin 1993 (> 20 years) | Nationality, age, sex, diagnoses, aetiology, degree of visual impairment, other impairments | Visual acuity < or = 6/60. | Among older subjects(60 years+): Age-related macular degeneration (71.4%), diabetic retinopathy (8.4%), glaucoma (5.0%). Among younger subjects (133 persons) aged 20-59 years: diabetic retinopathy 36%,lesions of the optic pathways 26%, myopia and retinitis pigmentosa 5% each. |
| Norway | Riise R. Nordic registers of visually impaired children. *Scand J Soc Med.* 1993;21(2):66-68 | 1992 | Prevalence: 15 per 100,000 children <17 years | 2527 children in Denmark, Norway, Iceland, FInland | Sex, year of birth, year of registration, classification of visual impairment, ocular diagnosis, systemic diagnosis, aetiology and additional impairments | Best corrected visual acuity in the best eye less than 6/18 or visual field less than 10 degrees around fixation | Optic nerve atrophy, cerebral amblyopia, retinopathy of prematurity |
| Iceland | Riise R. Nordic registers of visually impaired children. *Scand J Soc Med.* 1993;21(2):66-68 | 1992 | Prevalence: 19 per 100,000 children <17 years | 2527 children in Denmark, Norway, Iceland, FInland | Sex, year of birth, year of registration, classification of visual impairment, ocular diagnosis, systemic diagnosis, aetiology and evt. additional impairments | Best corrected visual acuity in the best eye less than 3/60 or visual field less than 10 degrees around fixation | Optic nerve atrophy, cerebral amblyopia, retinopathy of prematurity |
| Finland | Riise R. Nordic registers of visually impaired children. *Scand J Soc Med.* 1993;21(2):66-68 | 1992 | Prevalence 15 per 100,000 children <17 years | 2527 children in Denmark, Norway, Iceland, FInland | Sex, year of birth, year of registration, classification of visual impairment, ocular diagnosis, systemic diagnosis, aetiology and evt. additional impairments | Best corrected visual acuity in the best eye less than 3/60 or visual field less than 10 degrees around fixation | Optic nerve atrophy, cerebral amblyopia, retinopathy of prematurity |
| Germany | Rohrschneider K. [Blindness in Germany - comparison between retrospective data and predictions for the future]. *Der Ophthalmologe : Zeitschrift der Deutschen Ophthalmologischen Gesellschaft.* 2012;109(4):369-376 | 2009 | Prevalence 9.7 per 10,000 in 2009 | Estimated | Demographics, cause of blindness, visual acuity, visual field | Not reported | Age related macular degeneration |
| Sweden | Blohme J, Tornqvist K. Visual impairment in Swedish children. I. Register and prevalence data. *Acta ophthalmologica Scandinavica.* 1997;75(2):194-198.  Blohme J, Tornqvist K. Visual impairment in Swedish children. III. Diagnoses. *Acta ophthalmologica Scandinavica.* 1997;75(6):681-687 | 1994 | Prevalence: 109/100,000 aged 0 to 19 years | 2373  (0 to 19 years) | Demographics, cause of blindness, systemic diagnosis, classification of visual impairment, aetiology, additional impairments | Best corrected visual acuity in the best eye less than 6/18 (WHO categories including blindness best corrected visual acuity in the best eye less than 3/60 or visual field less than 10 degrees around fixation) | Non-hereditary optic atrophy, cerebral visual impairment, retinopathy of prematurity. |
| Republic of Ireland | Munier A, Gunning T, Kenny D, O'Keefe M. Causes of blindness in the adult population of the Republic of Ireland. *The British journal of ophthalmology.* 1998;82(6):630-633 | 1996 | Not stated | 5,002 (>16 years) | Demographics, cause of blindness, systemic diagnosis, visual acuity | Best corrected visual acuity of 6/60 (0.1) or less in the better eye or a visual field restricted to 20 degrees or less | Macular degeneration (16%), glaucoma (16%), cataract (11%), diabetic retinopathy (3%) |
| Republic of Ireland | Kelliher C, Kenny D, O'Brien C. Trends in blind registration in the adult population of the Republic of Ireland 1996-2003. *The British journal of ophthalmology.* 2006;90(3):367-371. | 2003 | Not stated | 6,862 (> 16 years) | Demographics, cause of vision impairment, blind registration condition, reasons for non-registration, permanence of vision loss | Best corrected visual acuity of 6/60 or less in the better eye, or a visual field subtending an angle of 20 degrees or less | Age related macular degeneration (25%), glaucoma (12%), retinitis pigmentosa (7%) |
| Northern Ireland | Canavan YM, Jackson AJ, Stewart A. Visual impairment in Northern Ireland. *The Ulster medical journal.* 1997;66(2):92-95 | 1996 | 0.35% of residents in Northern Ireland | 5,764. New cases added per years increased from 361 in 1984, to 731 in 1996 | Demographics, cause of blindness, visual acuity, visual field | Blindness: best corrected visual acuity of less than 3/60, or central visual field less than 10 degrees around fixation.  Low vision: best corrected visual acuity of 6/18 – 3/60 in better eye | Age-related macular degeneration, primary open angle glaucoma, diabetic retinopathy, myopia, senile cataract |
| Northern Ireland | Shirley K, Chamney S, Satkurunathan P, McLoone S, McLoone E, Medscape. Impact of healthcare strategies on patterns of paediatric sight impairment in a developed population: 1984-2011. *Eye (Lond).* 2017;31(11):1537-1545. | 1984 to 2011 | Not stated | 598 (<16 years old) | Demographics, cause, timing of insult, visual acuity | Severe sight impairment: Snellen visual acuity <3/60, or visual acuity between 3/60 and 6/60 with a very contracted field of vision, or visual acuity >6/60 but contracted field of vision especially inferiorly  Sight impairment: Snellen visual acuity between 3/60 and 6/60 with full field, visual acuity up to 6/24 with moderate field contraction, opacities in the media or aphakia, or visual acuity of 6/18 or better with marked field contraction | Optic atrophy (16%), albinism (12%), cerebral visial impairment (11%), congenital cataract (8%), retinopathy of prematurity (8%), congenital motor nystagmus (7%) |
| England | Brennan ME, Knox EG. An investigation into the purposes, accuracy, and effective uses of the Blind Register in England. *British journal of preventive & social medicine.* 1973;27(3):154-159 | 1967 | 207 per 100,000 | 102,597 (all ages) | Demographics, marital status, current and previous employment, comorbidities, family history, prognosis, | Visual acuity below 3/60 Snellen in both eyes, or visual acuity 3/60 but less than 6/60 if field of vision considerably constricted | Not reported |
| England and Wales | Bunce C, Wormald R. Causes of blind certifications in England and Wales: April 1999-March 2000. *Eye (Lond).* 2008;22(7):905-911. | 1999-2000 | Not stated | 34,410 newly registered (all ages) | Demographics, cause of blindness, visual acuity, visual field | Best corrected visual acuity below 3/60, or below 6/60 with very constricted field of vision, or 6/60 or above with very constricted field of vision, especially in lower part of field | Age-related macular degeneration (57.2%), glaucoma (10.9%), diabetic retinopathy (5.9%), optic atrophy (3.1%), cardiovascular disease/accidents (2.5%) |
| England and Wales | Bunce C, Xing W, Wormald R. Causes of blind and partial sight certifications in England and Wales: April 2007-March 2008. *Eye (Lond).* 2010;24(11):1692-1699 | 2007-2008 | Not stated | 23,185 newly registered (all ages) | Demographics, cause of blindness, visual acuity, visual field | Severely sight impaired: best corrected visual acuity less than 3/60, or between 3/60 – 6/60 with severe reduction of field, or 6/60 or above but with very reduced field of vision.  Sight impaired: best corrected visual acuity 3/60 – 6/60, or between 6/60 – 6/24 with moderate of reduction of field, or 6/18 or better with large field defect | Degeneration of the retina and posterior pole (58.6%), glaucoma (6.3%), hereditary retinal disorders (5.5%), optic atrophy (4.2%) |
| England | Malik AN, Bunce C, Wormald R, Suleman M, Stratton I, Gray JA. Geographical variation in certification rates of blindness and sight impairment in England, 2008-2009. *BMJ open.* 2012;2(6). | 2008-2009 | Not stated | 23,773 newly registered (all ages) | Demographics, cause of blindness, visual acuity, visual field | Severely sight impaired: best corrected visual acuity less than 3/60, or between 3/60 – 6/60 with severe reduction of field, or 6/60 or above but with very reduced field of vision.  Sight impaired: best corrected visual acuity 3/60 – 6/60, or between 6/60 – 6/24 with moderate of reduction of field, or 6/18 or better with large field defect | Not reported |
| England and Wales | Liew G, Michaelides M, Bunce C. A comparison of the causes of blindness certifications in England and Wales in working age adults (16-64 years), 1999-2000 with 2009-2010. *BMJ open.* 2014;4(2):e004015 | 1999-2000 and  2009-2010 | Not stated (up to 53% believed eligible not registered) | 1,637 newly registered  1756 newly registered (16-64 years) | Demographics, cause of blindness, visual acuity, visual field | Severely sight impaired: best corrected visual acuity less than 3/60, or between 3/60 – 6/60 with severe reduction of field, or 6/60 or above but with very reduced field of vision.  Sight impaired: best corrected visual acuity 3/60 – 6/60, or between 6/60 – 6/24 with moderate of reduction of field, or 6/18 or better with large field defect | From 2009 to 2010: Hereditary retinal disorders (20.2%), diabetic retinopathy/maculopathy (14.4%), optic atrophy (14.1%)  From 1999 to 2000: Diabetic retinopathy/maculopathy (17.7%), hereditary retinal disorders (15.8%), optic atrophy (10.1%) |
| England and Wales | Mitry D, Bunce C, Wormald R, et al. Causes of certifications for severe sight impairment (blind) and sight impairment (partial sight) in children in England and Wales. *The British journal of ophthalmology.* 2013;97(11):1431-1436. | 1999-2000 and  2009-2010 | Not stated | 861 in 1999-2000, 1040 In 2009-2010 (</= 16 years old) | Demographics, cause of blindness, other ocular diagnoses | Severely sight impaired: best corrected visual acuity less than 3/60, or between 3/60 – 6/60 with severe reduction of field, or 6/60 or above but with very reduced field of vision.  Sight impaired: best corrected visual acuity 3/60 – 6/60, or between 6/60 – 6/24 with moderate of reduction of field, or 6/18 or better with large field defect | From 1999-2000: SSI - cerebral visual impairment (23.2%), optic nerve disorders (23.2%). SI – nystagmus (16.7%), optic nerve disorders (15.5%)  From 2007-2008: SSI – cerebral visual impairment (21%)  From 2009-2010: SSI cerebral visual impairment (31%). SI – congenital globe anomalies (18.4%), retinal dystrophy (16.6%) |
| England | Mitry D, Bunce C, Wormald R, Bowman R. Childhood visual impairment in England: a rising trend. *Archives of disease in childhood.* 2013;98(5):378-380. | 2011 | Not stated | Incidence: 0.41 per 100,000 (children) | Demographics, visual acuity, visual field | Severely sight impaired: best corrected visual acuity less than 3/60, or between 3/60 – 6/60 with severe reduction of field, or 6/60 or above but with very reduced field of vision.  Sight impaired: best corrected visual acuity 3/60 – 6/60, or between 6/60 – 6/24 with moderate of reduction of field, or 6/18 or better with large field defect | Not reported |
| England and Wales | Quartilho A, Simkiss P, Zekite A, Xing W, Wormald R, Bunce C. Leading causes of certifiable visual loss in England and Wales during the year ending 31 March 2013. *Eye (Lond).* 2016;30(4):602-607 | 2007-2008  and  2012-2013 | Not stated | 13,530 in 2007-2008  and  24,009 in 2012-2013 | Demographics, cause, timing of insult, visual acuity | Severely sight impaired: best corrected visual acuity less than 3/60, or between 3/60 – 6/60 with severe reduction of field, or 6/60 or above but with very reduced field of vision.  Sight impaired: best corrected visual acuity 3/60 – 6/60, or between 6/60 – 6/24 with moderate of reduction of field, or 6/18 or better with large field defect | Severe sight impaired – degeneration of the macula and posterior pole (50%), glaucoma 11%), hereditary retinal disorders (8.2%), diabetic retinopathy (6.3%), optic atrophy (4.9%), cerebrovascular disease (2.7%), visual cortex disorder (2.6%), congenital anomalies (2.1%), retinal vascular occlusion (2%) |
| England and Wales | Bunce C, Zekite A, Wormald R, Bowman R. Is there evidence that the yearly numbers of children newly certified with sight impairment in England and Wales has increased between 1999/2000 and 2014/2015? A cross-sectional study. *BMJ open.* 2017;7(9):e016888. | 1999-2000  and  2014-2015 | Incidence: 8.2 per 100,000 children in 1999/200, and 13.3 per 100,000 children in 2014/2015 (< 16 years) | 472 newly registered in 2000; 859 newly registered in 2015 | Demographics, age at certification, cause of blindness, visual acuity, visual field | Severely sight impaired: best corrected visual acuity less than 3/60, or between 3/60 – 6/60 with severe reduction of field, or 6/60 or above but with very reduced field of vision.  Sight impaired: best corrected visual acuity 3/60 – 6/60, or between 6/60 – 6/24 with moderate of reduction of field, or 6/18 or better with large field defect | 2014/2015 causes of severe sight impairment: Hereditary retinal dystrophies (16%), cerebral visual impairment (15%), optic nerve and visual pathway disorders (11%), congenital anomalies (11%), albinism (4%), retinopathy of prematurity (3%) |
| England and Wales | Rahman F, Zekite A, Bunce C, Jayaram H, Flanagan D. Recent trends in vision impairment certifications in England and Wales. *Eye (Lond).* 2020;34(7):1271-1278. | 2018 | Not stated | Incidence 41/100,000 | Total registrations, registrations due to preventable sight loss | Severely sight impaired: best corrected visual acuity less than 3/60, or between 3/60 – 6/60 with severe reduction of field, or 6/60 or above but with very reduced field of vision.  Sight impaired: best corrected visual acuity 3/60 – 6/60, or between 6/60 – 6/24 with moderate of reduction of field, or 6/18 or better with large field defect | Severe sight impairment - Hereditary retinal dystrophy, disorders of the visual cortex and brain, albinism  Sight impairment – Hereditary retinal dystrophy, nystagmus and other irregular eye movements, albinism |
| Israel | Hod Y, Corcia Y, Yassur Y, Geyer O. [Causes of blindness in Israel]. *Harefuah.* 2000;138(4):276-278, 342 | 1998 | Not stated | 15,937 (all ages) | Demographics, cause of blindness | Visual acuity </= 0.05 (20/400) or a visual field of <20 degrees radius in better eye | Glaucoma (13%), macular degeneration (12%), diabetic eye disease (11%) |
| Israel | Farber MD. National Registry for the Blind in Israel: estimation of prevalence and incidence rates and causes of blindness. *Ophthalmic epidemiology.* 2003;10(4):267-277. | 1987-1999 | Prevalence 0.31%, incidence 0.037% | 18,891 registered between 1987-1999.  2,511 new registrations in 1999 (all ages) | Demographics, visual acuity, visual field loss, cause of blindness | Visual acuity </= 0.05 (20/400) or a visual field of <20 degrees radius in better eye^1-3^ | Age-related macular degeneration (14%), glaucoma (14%), diabetic retinopathy (11%), cataract (10%), myopic maculopathy (10%), optic atrophy (8.4%) |
| Israel | Avisar R, Bahar I, Weinberger D. [Causes of blindness in the year 2000 in Israel]. *Harefuah.* 2003;142(2):94-96, 160. | 2000 | Not stated | 15,937 (all ages) | Demographics, cause of blindness, visual acuity, visual field | Visual acuity </= 0.05 (20/400) or a visual field of <20 degrees radius in better eye | Macular degeneration, glaucoma, diabetic eye disease |
| Israel | Merrick J, Bergwerk K, Morad M, Carmeli E. Blindness in adolescents in Israel. *Int J Adolesc Med Health.* 2004;16(1):79-81 | 2000 | Not stated | 18,509 (all ages) | Demographics, cause of blindness, visual acuity, visual field | Visual acuity </= 0.05 (20/400) or a visual field of <20 degrees radius in better eye | Optic atrophy |
| Israel | Avisar R, Friling R, Snir M, Avisar I, Weinberger D. Estimation of prevalence and incidence rates and causes of blindness in Israel, 1998-2003. *The Israel Medical Association journal : IMAJ.* 2006;8(12):880-881. | 1998 and  2003 | Not stated | 21,585 (all ages) | Demographics, cause of blindness, visual acuity, visual field | Visual acuity </= 0.05 (20/400) or a visual field of <20 degrees radius in better eye | In 1998: Age-related macular degeneration (20.1%), glaucoma (13.8%), myopic maculopathy (10.1%), cataract (10.4%), diabetic retinopathy/ maculopathy (10.1%), optic atrophy (7.9%).  In 2003: Age-related macular degeneration (28%), diabetic retinopathy/maculopathy (14.4%), glaucoma (11.8%), myopic maculopathy (7.4%), cataract (6.5%), optic atrophy (6.5%) |
| Israel | Skaat A, Chetrit A, Belkin M, Kinori M, Kalter-Leibovici O. Time trends in the incidence and causes of blindness in Israel. *American journal of ophthalmology.* 2012;153(2):214-221 e211. | 1999 and  2008 | Incidence 16.6 per 100,000 | 19,862 new registrations between 1999-2008 (all ages) | Demographics, cause of blindness, visual acuity, visual field, year of registration | Blindness: best corrected visual acuity less than 1/60 or central visual field not more than 10 degrees in better eye  Severe visual impairment: visual acuity less than 3/60 but 1/60 or better, or visual field of less than 20 degrees in better eye | In 1999: age-related macular degeneration (20.9%), diabetic retinopathy (12.5%), glaucoma 13.9%), cataract (9.8%).  In 2008: age-related macular degeneration (28.7%), diabetic retinopathy (13.2%), glaucoma (12.1%), cataract (5.1%) |
| Israel | Mezer E, Chetrit A, Kalter-Leibovici O, Kinori M, Ben-Zion I, Wygnanski-Jaffe T. Trends in the incidence and causes of severe visual impairment and blindness in children from Israel. *Journal of AAPOS : the official publication of the American Association for Pediatric Ophthalmology and Strabismus.* 2015;19(3):260-265 e261. | 2013 | Incidence 3.05 per 100,000 children | 83 new registrations in 2013 (<18 years) | Demographics, age at registration, cause of blindness, visual acuity, visual field | Visual acuity </= 0.05 (20/400) or a visual field of <20 degrees radius in better eye | Optic atrophy (13%), retinitis pigmentosa (13%), retinopathy of prematurity (4%), albinism (4%) |

Note on literature search methods: Studies were identified from Pubmed search initially run in 2016 and updated to 5/7/23. We screened the titles and abstracts of 310 studies identifed using the following search strategy, and obtained full text for the 35 studies included in the above table:

((((("blind register"[All Fields] OR "blind registration"[All Fields] OR "blind registrations"[All Fields] OR "blind registry"[All Fields]) OR ("certification of blindness"[All Fields])) OR ("sight impairment certification"[All Fields] OR "sight impairment registration"[All Fields])) OR ("partial sight registers"[All Fields] OR "partial sight registration"[All Fields])) OR ("certifiable visual impairment"[All Fields] OR "certifiable visual loss"[All Fields])) OR (certifiable sight)

Our Pubmed literature search to June 2023 identified no studies from Sub-saharan Africa, South Asia, Central Europe, Eastern Europe and Central Asia, or Southeast Asia, East Asia and Oceania.
