# Supplementary material for: Sight impairment registration in Trinidad: trend in causes and population coverage in comparison to the National Eye Survey of Trinidad and Tobago
Source: Eye (Lond). 2024 Feb 7;38(11):2134–42. doi: 10.1038/s41433-024-02943-3 (PMC11269719; doi:10.1038/s41433-024-02943-3)
Supplement: Supplementary file 2 — Supplementary Tables 2–7 [file 41433_2024_2943_MOESM2_ESM.docx]

**Supplementary Tables**

**Supplementary Table 2: Benefits associated with TTBWA registration in 2016**

| **Benefits of TTBWA registration** |
| --- |
| In 2016, the TTBWA branches in Port-of-Spain and San Fernando, Trinidad, offered classes and services, via local libraries or home visits. These included:   - leisure activities; - mobility and orientation training, including provision of white sticks/canes; - daily living skills, including self-care, washing clothes and using the stove; - social and communication skills training, including Braille classes and courses on using the computer and assistive technology. This training took place at 3 different locations weekly, with about 10 people attending per location; - a socialisation programme including a board game club and client empowerment group; - vocational counselling and training in massage therapy and basket weaving; - counselling services for people recently diagnosed as visually impaired or blind, and their families; - social welfare assistance.   All people certified as visually impaired or blind were eligible to apply for a disability grant and social welfare from the Ministry of Social Development and Family Services.  TTBWA clients were able to access the sole low vision clinic in Trinidad and Tobago, established by the University of the West Indies’ School of Optometry in St. Augustine in 2012, where low vision aids and devices were available, with the cost of this funded directly by the TTBWA. |

**Supplementary Table 3: Standardised Recruitment approach for TTBWA validation study**

| **Recruitment process** |
| --- |
| 1. Staff in the TTBWA Headquarters contacted clients by telephone to explain purpose of the research study and the outcome of the call was recorded as “willing to participate”, “unwilling to participate” (reason noted), “deceased” or “no contact”. 2. If no telephone number was available on the register, or if the number was incorrect or out of service, staff used the Trinidad and Tobago Residential Directory to look up the client using both name and address. 3. Active telephone numbers were called up to 3 times at different times on different days, and a message was left if an answering machine was available, giving a short explanation about the study and a contact number. 4. Participants were offered free transport from the TTBWA Headquarters in Port of Spain (Northwest Trinidad) to an appointment at the research clinic in the University of the West Indies Department of Optometry, St Augustine (Northcentral Trinidad). |

**Supplementary Table 4: Characteristics of clients on the 2016 TTBWA Register in Trinidad, and comparison of responders and non-responders**

|  |  | All | Responders | Non-responders | OR (95% CI) | p-value |
| --- | --- | --- | --- | --- | --- | --- |
| n |  | 863 | 148 | 715 |  |  |
| Address recorded | Yes, n (%) | 853 (98.8) | 146 (98.7) | 707 (98.9) |  |  |
| Telephone recorded | Yes, n (%) | 478 (55.4) | 148 (100) | 380 (53.2) |  |  |
| Age | Mean (sd) | 62.1 (20.7) | 53.0 (20.3) | 64.0 (20.3) | 0.98 (0.97 to 0.99) | 0.0004 |
|  | Range | 2 to 107 | 12 to 97 | 2 to 107 |  |  |
| Age at registration | Mean (sd) | 47.6 (22.2) | 48.6 (21.2) | 47.3 (22.4) |  |  |
|  | Range | 0 to 96 | 0 to 89 | 0 to 96 |  |  |
| Gender | Male, n(%) | 415 (48.1) | 67 (45.3) | 350 (49.0) | 1.22 (0.86 to 1.75) | 0.2641** |
| Years since | median (IQR) | 12 (9 to 20) | 9 (4 to 17.5) | 13 (9 to 20) | Non-linear |  |
| registration | range | 1 to 91 | 1 to 52 | 1 to 91 |  |  |
|  | 0 to 10 years |  |  |  | 1 | <0.0001 |
|  | 11 to 20 years |  |  |  | 0.25 (0.14 to 0.43) |  |
|  | 21 to 30 years |  |  |  | 0.38 (0.21 to 0.69) |  |
|  | > 31 years |  |  |  | 0.46 (0.23 to 0.91) |  |
| Region | Northwest | 244 (28.3) | 73 (49.3) | 171 (23.9) | 1 | <0.0001 |
|  | Northcentral | 215 (24.9) | 48 (32.4) | 167 (23.4) | 0.46 (0.28 to 0.76) |  |
|  | East | 59 (6.8) | 5 (3.4) | 54 (7.6) | 0.17 (0.10 to 0.31) |  |
|  | South | 341 (39.5) | 22 (14.9) | 319 (44.6) | 0.18 (0.06 to 0.53) |  |
| Marital | Single | 355 (41.1) | 83 (56.1) | 272 (38.0) | 1 | 0.1201 |
| status | Married | 304 (35.2) | 38 (25.7) | 266 (37.2) | 0.61 (0.37 to 1.00) |  |
|  | Separated | 13 (1.5) | 1 (0.7) | 12 (1.7) | 0.44 (0.05 to 3.87) |  |
|  | Widowed | 94 (10.9) | 7 (4.7) | 87 (12.2) | 0.51 (0.20 to 1.28) |  |
|  | Divorced | 23 (2.7) | 7 (4.7) | 16 (2.2) | 1.87 (0.66 to 5.29) |  |
|  | Cohabiting | 10 (1.2) | 2 (1.4) | 8 (1.1) | 0.41 (0.07 to 2.27) |  |
|  | Not reported | 64 (7.4) | 10 (6.8) | 54 (7.6) | 0.50 (0.21 to 1.21) |  |
| Causes | Unknown | 157 (18.2) | 31 (21.0) | 126(17.6) | 1 | 0.0918 |
|  | Trauma | 67 (7.8) | 11 (7.4) | 56(7.8) | 0.59 (0.25 to 1.42) |  |
|  | Cataract | 99 (11.5) | 11 (7.4) | 88 (12.3) | 0.42 (0.18 to 0.96) |  |
|  | Congenital cataract | 3 (0.4) | 2 (1.4) | 1 (0.1) | 6.39 (0.48 to 85.86) |  |
|  | Corneal pathology | 5 (0.6) | 1 (0.7) | 4 (0.6) | 0.58 (0.05 to 6.62) |  |
|  | Congenital | 45 (5.2) | 9 (6.1) | 36 (5.0) | 1.05 (0.40 to 2.80) |  |
|  | Diabetes | 82 (9.5) | 6 (4.1) | 76 (10.6) | 0.28 (0.10 to 0.77) |  |
|  | Diabetes + Glaucoma | 18 (2.1) | 6 (4.1) | 12 (1.7) | 1.75 (0.53 to 5.72) |  |
|  | Glaucoma | 225 (26.1) | 33 (22.3) | 192 (26.9) | 0.66 (0.35 to 1.26) |  |
|  | Optic nerve pathology | 9 (1.0) | 2 (1.4) | 7 (1.0) | 0.99 (0.15 to 6.77) |  |
|  | Refractive/amblyopia | 3 (0.4) | 2 (1.4) | 1 (0.1) | 5.70 (0.27 to 120.88) |  |
|  | ROP | 11 (1.3) | 7 (4.7) | 4 (0.6) | 3.66 (0.86 to 15.56) |  |
|  | Intracranial | 9 (1.0) | 2 (1.4) | 7 (1.0) | 0.62 (0.08 to 4.79) |  |
|  | Other | 8 (0.9) | 1 (0.7) | 7 (1.0) | 0.56 (0.06 to 5.43) |  |
|  | Drugs or nutritional | 2 (0.2) | 1 (0.7) | 1 (0.1) | not estimable |  |
|  | Retinal detachment | 36 (4.2) | 9 (6.1) | 27 (3.8) | 1.32 (0.49 to 3.55) |  |
|  | Macular degeneration | 46 (5.3) | 4 (2.7) | 42 (5.9) | 0.74 (0.21 to 2.61) |  |
|  | Retinal dystrophy | 23 (2.7) | 6 (4.1) | 17 (2.4) | 1.10 (0.36 to 3.36) |  |
|  | Infectious | 3 (0.4) | 1 (0.7) | 2 (0.3) | 1.26 (0.09 to 17.22) |  |
|  | Inflammatory | 4 (0.5) | 1 (0.7) | 3 (0.4) | 0.61 (0.05 to 8.04) |  |
|  | Ocular cancer | 8 (0.9) | 2 (1.4) | 6 (0.8) | 0.97 (0.16 to 5.82) |  |

*Odds ratios (OR) from the multiple logistic regression model (Model), adjusted for variables that were significant in the single variable analysis [age (linear), marital status, region, causes, and years register]. Global p value from likelihood-ratio test (LRT).

** OR for gender from single variable analysis.

**Supplementary Table 5: Narrative examples of causes of potentially avoidable vision loss identified by the study ophthalmologist following examination of a subgroup of TTBWA clients in July-August 2013**

| Examples of causes of potentially avoidable vision loss |
| --- |
| Adults examined (n=71) |
| • Late presentation of advanced glaucoma  • Poor compliance with intraocular pressure lowering medication for glaucoma  • Suboptimal primary and secondary prevention in diabetic patients  • Ocular injuries arising from failure to wear protective goggles at work  • Ocular injuries arising from non-accidental injury including domestic violence  • Suboptimal availability of timely vitreoretinal surgery for retinal detachment  • Uncorrected refractive error resulting from unaffordability of spectacles  • Suboptimal antenatal care to detect pre-eclampsia and prevent eclampsia  • Unavailability of screening and treatment for retinopathy of prematurity |
| Children examined (n=16) |
| • Retinopathy of prematurity: no ROP screening programme, delayed diagnosis of ROP, delayed laser therapy for ROP treatment, with total retinal detachment  • Unavailability of timely vitreoretinal surgery for retinal detachment  • Unavailability of timely cataract extraction for congenital cataract  • Delayed diagnosis of amblyopia, and no access to orthoptic support or occlusion therapy |

**Supplementary Table 6: Narrative examples of TTBWA client feedback on eye care services in Trinidad and Tobago (July-August 2013)**

| Survey question | Narrative summary of responses |
| --- | --- |
| “Is there anything you would like to change about the current eye care system in Trinidad?” | • More time during appointments with the doctor; better explanations to the  patient  • Increased availability of diagnostic tests and equipment in the hospital eye  clinics  • Reduced waiting time before first visit to eye clinic, and before treatment and  surgery  • Better clinic organization  • Cheaper access to eye drops  • Eye screening for babies born prematurely  • Educate the public on diet and causes of blindness  • Research on vision loss  • Better training of the doctors, more specialists  • Better maintenance of public hospital equipment (e.g. the laser)  • Subsidised cost of sight tests and spectacles for those in need |

**Supplementary Table 7: Comparison of benefits of blind registration in Trinidad and Tobago in 2016, with three other countries (United Kingdom, a state in the United States, and Israel).**

| Benefit | Trinidad and Tobago  (2016) | United Kingdom  (2021)^44^ | United States of America (Massachusetts)  (2023)^47^ | Israel  (2022)^48^ |
| --- | --- | --- | --- | --- |
| Access to low vision clinic at no cost to individual | Yes | Yes | Yes | Yes |
| Education support (e.g. School for the Blind) | Yes | Yes | Yes | Yes |
| Disability grant from the Government | Yes | Yes | Yes | Yes |
| Social welfare grant | Yes | Yes | Yes | Yes |
| Real estate tax exemption | No | No | Yes | No |
| Carer’s allowance | No | Yes | No | No |
| Automobile tax exemption | No | No | Yes | No |
| Public transport concession | No | Yes | Yes | Yes |
| Handicap parking | No | Yes | Yes | Yes |
| Communication equipment | No | Yes | Yes | Yes |
| Communication training (e.g. Braille) | Yes | Yes | Yes | Yes |
| Mobility and orientation training (e.g. access to a white cane) | Yes | Yes | Yes | Yes |
| Socialisation and leisure activities | Yes | Yes | Yes | Yes |
| Vocational training | Yes | Yes | Yes | Yes |
